# Bone Marrow Mesenchymal Stem Cell-Derived Exosomes Modulate Chemoradiotherapy Response in Cervical Cancer Spheroids

**DOI:** 10.3390/ph18071050

**Published:** 2025-07-17

**Authors:** Kesara Nittayaboon, Piyatida Molika, Rassanee Bissanum, Kittinun Leetanaporn, Nipha Chumsuwan, Raphatphorn Navakanitworakul

**Affiliations:** 1 Department of Biomedical Sciences and Biomedical Engineering, Faculty of Medicine, Prince of Songkla University, Hat Yai 90110, Thailand; nkesara@medicine.psu.ac.th (K.N.); 6310330014@email.psu.ac.th (P.M.); rassanee.b@psu.ac.th (R.B.); kittinun.l@psu.ac.th (K.L.); 2 Translational Medicine Research Center, Faculty of Medicine, Prince of Songkla University, Hat Yai 90110, Thailand; 3 Department of Radiology, Faculty of Medicine, Prince of Songkla University, Hat Yai 90110, Thailand; cnipha@medicine.psu.ac.th; 4 Division of Therapeutic Radiology and Oncology, Department of Radiology, Faculty of Medicine, Prince of Songkla University, Hat Yai 90110, Thailand

**Keywords:** bone marrow mesenchymal stem cells (BM-MSCs), exosomes, cervical cancer, cancer therapy, chemo- and radiotherapy resistance

## Abstract

**Background:** Bone marrow mesenchymal stem cells (BM-MSCs) are significant in chemo- and radiotherapy resistance. Previous research has focused on BM-MSCs, demonstrating their functional involvement in cancer progression as mediators in the tumor microenvironment. They play multiple roles in tumorigenesis, angiogenesis, and metastasis. BM-MSC-derived exosomes (BM-MSCs-exo) are small vesicles, typically 50–300 nm in diameter, isolated from BM-MSCs. Some studies have demonstrated the tumor-suppressive effects of BM-MSCs-exo. **Objective:** This study aimed to investigate their role in modulating the impact of chemoradiotherapy (CRT) in different types of cervical cancer spheroid cells. **Methods:** The spheroids after treatment were subject to size measurement, cell viability, and caspase activity. Then, the molecular mechanism was elucidated by Western blot analysis. **Results:** We observed a reduction in spheroid size and an increase in cell death in HeLa spheroids, while no significant changes in size or cell viability were found in SiHa spheroids. At the molecular level, CRT treatment combined with BM-MSCs-exo in HeLa spheroids induced apoptosis through the activation of the NF-κB pathway, specifically via the NF-κB1 (P50) transcription factor, leading to the upregulation of apoptosis-related molecules. In contrast, CRT combined with BM-MSCs-exo in SiHa spheroids exhibited an opposing effect: although cellular viability decreased, caspase activity also decreased, which correlated with increased HSP27 expression and the subsequent downregulation of apoptotic molecules. **Conclusion:** Our study provides deeper insight into the potential of BM-MSCs-exo in cervical cancer treatment, supporting the development of more effective and safer therapeutic strategies for clinical application.

## 1. Introduction

Mesenchymal stem cells (MSCs) have self-renewal and multilineage differentiation capabilities and can be derived from several sources, including dental tissue, adipose tissue, the umbilical cord, and bone marrow. Previous studies have shown that MSCs play important roles in both physiological and pathological conditions [1,2]. Bone marrow-derived mesenchymal stem cells (BM-MSCs) may participate in the modulation of the tumor microenvironment, including immune system regulation and tumor growth. Several studies have demonstrated that BM-MSCs contribute to cancer cell resistance against anti-cancer drugs through anti-apoptotic activity and the stimulation of cellular growth [3]. MSC-derived exosomes (MSCs-exo) have also been implicated in cancer progression, therapy response, and chemotherapy resistance. These exosomes directly deliver RNAs and proteins involved in drug resistance processes across various cancer types. The cargoes in MSCs-exo are known to modulate apoptosis-related proteins, thereby reducing chemotherapy responsiveness in cancer cells. A previous study of breast cancer suggested that human BM-MSCs-exo promote tumor growth via miRNAs, such as miR-21 and miR-34a, and the activation of the VEGF/ERK pathway [4,5].

Chemotherapy and radiotherapy are key strategies in the treatment of cervical cancer. Radiation therapy is widely used in cancers such as those affecting the prostate, lung, and head and neck and is applied in approximately 50% of all cancer cases, contributing to about 40% of curative treatments [6,7]. However, cancer patients often develop radioresistance due to complex mechanisms, limiting therapeutic efficacy and contributing to metastasis and recurrence [8]. In gastric cancer, MSCs-exo have been shown to enhance resistance to 5-FU via the activation of the Raf/Mek/ERK pathway [5]. Additionally, MSCs-exo have been found to increase resistance to carboplatin and doxorubicin in breast cancer by transferring miR-222/223 and miR-236, respectively [9]. On the other hand, MSCs-exo have been shown to enhance the therapeutic response to doxorubicin and paclitaxel in ovarian and hepatocellular carcinoma, respectively [10]. Key molecules involved include miR-146a, which affects the LAMC2/PI3K/Akt pathway [11], and miR-451a, which downregulates ADAM10 expression and enhances drug response in vitro [12,13]. In terms of radiotherapy resistance, MSCs-exo carrying miR-340-5p have been shown to suppress radiation-induced apoptosis and promote DNA damage repair via KLF10 in hypoxic esophageal squamous cell carcinoma models [14]. Conversely, exosomes overexpressing miR-34c have demonstrated the ability to enhance radiation-induced apoptosis in nasopharyngeal carcinoma [15].

**Figure 1:**
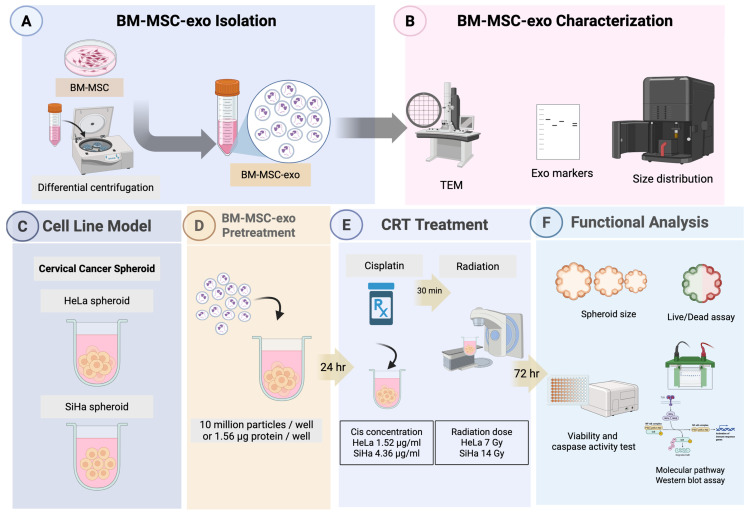
Research workflow. This flowchart represents the experimental design. BM-MSCs-exo were isolated and characterized (**A**,**B**). Cervical spheroid cell cultures (**C**) were pre-treated (**D**) using BM-MSCs-exo, and then, CRT treatment was performed (**E**). After all treatment, the spheroids were incubated for 72 h, and functional analysis was then conducted (**F**). BM-MSC: Bone marrow mesenchymal stem cells; BM-MSC-exo: Bone marrow mesenchymal stem cell-derived exosome; Exo: Exosome; TEM: Transmission electron microscope; CRT: Chemoradiotherapy; Cis: Cisplatin; Gy: Gray. Created in BioRender. Nittayaboon, K. (2025).

In this study, we investigated the effect of natural cargo of BM-MSCs-exo on two cervical cancer spheroid models—HeLa and SiHa—which exhibited different cellular characteristics. The spheroids were pre-treated with or without BM-MSCs-exo, followed by chemoradiotherapy (CRT). Subsequently, viability and functional assays were performed to assess therapeutic response. Figure 1 presents the experimental design and workflow.

## 2. Results

### 2.1. Characterization and Quantification of Bone Marrow Mesenchymal Stem Cell-Derived Exosomes (BM-MSCs-Exo)

**Figure 2:**
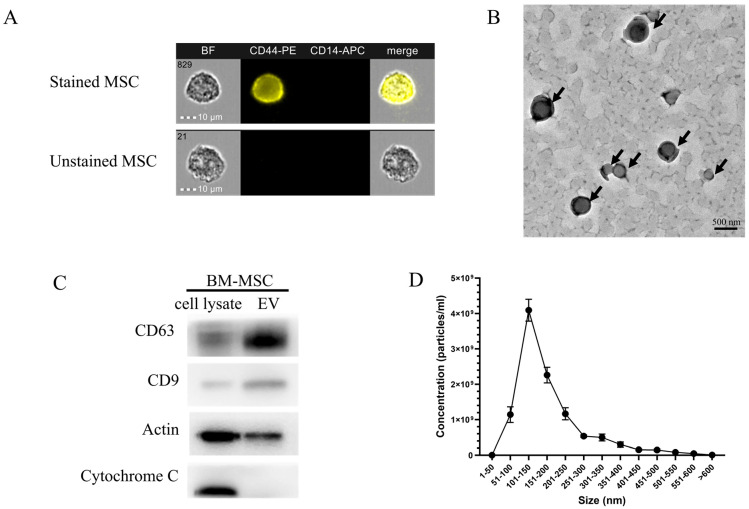
Characterization of BM-MSCs-exo (**A**). CD44-positive and CD14-negative cell surface marker staining indicated the presence of BM-MSCs (**B**). The transmission electron microscope (TEM) showed cup-shape morphology of the BM-MSCs-exo indicated by the black arrows (**C**). Western blot analysis showed the protein expression of EV markers, including CD63 and CD9. Actin was used as housekeeping protein, and Cytochrome C served to control EV purity (**D**). The size distribution of BM-MSCs-exo was analyzed using nanoparticle tracking analysis (NTA). BF: Bright field; CD44-PE; CD44 protein conjugated with the fluorescent molecule phycoerythrin; CD14-APC: CD14 protein conjugated with the fluorescent molecule allophycocyanin; BM-MSC-exo: Bone marrow mesenchymal stem cell-derived exosome; MSC: Mesenchymal stem cells; EV: Extracellular vesicle; BF: Bright field; CD44-PE; CD44 protein conjugated with the fluorescent molecule phycoerythrin; CD14-APC: CD14 protein conjugated with the fluorescent molecule allophycocyanin.

Exosome morphology and size were visualized using transmission electron microscopy (TEM) and nanoparticle tracking analysis (NTA). Additionally, exosome markers were detected using Western blot analysis (Figure 2). Figure 2A shows the morphology of BM-MSC-derived exosomes (BM-MSCs-exo). A cup-shaped, red blood cell-like structure was observed under TEM at 110,000× magnification, as indicated by the black arrows. The presence of extracellular vesicle (EV) markers, including CD63 and CD9, was strongly expressed in the EV fraction compared to in the cell lysate, as shown in Figure 2B. Actin was used as a housekeeping protein, while Cytochrome C served as a negative control, indicating no contamination from cellular components in the EV sample. The size distribution of the BM-MSCs-exo, ranging from 100 to 500 nm in diameter, was confirmed using NTA (Figure 2C). The mean particle size was 182.9 ± 2.5 nm, and the mode size was 110.0 ± 4.6 nm, indicating a heterogeneous vesicle population.

### 2.2. The Effect of Crt Combined with Bm-Mscs-Exo Pre-Treatment

#### 2.2.1. BM-MSCs-Exo Pre-Treatment Decreased the Size of Hela Spheroids but Had No Effect on SiHa Spheroids After CRT Treatment

The effect of BM-MSCs-exo on CRT treatment was evaluated via pre-treating cervical cancer spheroids with BM-MSCs-exo for 24 h prior to the administration of chemotherapy and radiotherapy. The spheroids were then cultured for 72 h, after which their size, viability, caspase activity, and molecular mechanisms were assessed. An overview of the experimental design is shown in Figure 1.

**Figure 3:**
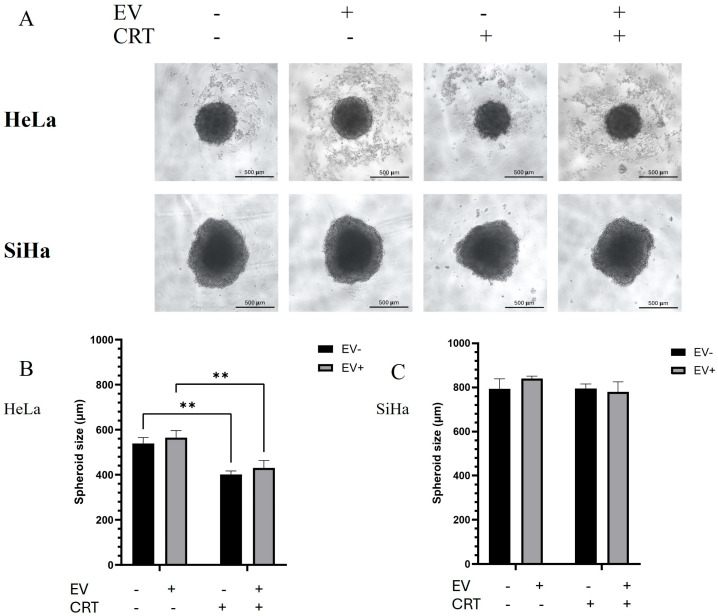
The effect of BM-MSCs-exo combined with CRT treatment on cervical cancer spheroids. CRT treatment resulted in a slight decrease in spheroid size in HeLa but not in SiHa spheroids (**A**). The morphology of HeLa (upper panel) and SiHa (lower panel) spheroids was captured using inverted microscopy at 10× magnification (scale bar = 500 µm). (**B**,**C**) The sizes of SiHa and HeLa spheroids were measured using ImageJ software. Three independent experiments were conducted. Student’s *t*-test was used to determine statistical significance (** *p* < 0.01). EV: Extracellular vesicle; CRT: Chemoradiotherapy.

The morphology of the spheroids under each condition was captured after 72 h of treatment. Spheroid size was analyzed by measuring the diameter of each spheroid using ImageJ software (Version 1.54m). Figure 3 illustrates the differences in spheroid morphology and size between the HeLa and SiHa spheroids. In the untreated control group, HeLa spheroids had an average diameter of 538.73 ± 21.31 µm. Treatment with BM-MSC-derived EVs alone led to an increase in spheroid size, with spheroids appearing larger than those in the control group, though this change was not statistically significant. In contrast, CRT treatment markedly reduced spheroid diameter to 401.03 ± 12.67 µm, indicating a significant anti-proliferative or cytotoxic effect. Pre-treatment with EVs followed by CRT (EV+CRT group) did not significantly alter spheroid size compared to CRT alone. Similarly, the size of SiHa spheroids did not significantly differ between the control and CRT-treated groups, with average sizes of 793.32 ± 36.91 µm and 794.93 ± 16.80 µm, respectively. This observation can be explained by differences in spheroid morphology. HeLa cells form compact spheroids, while SiHa cells tend to form looser, flatter spheroids [16,17]. After CRT treatment, the growth of compact HeLa spheroids was inhibited, resulting in no further size increase. In contrast, the loosely compacted SiHa spheroids had already reached a flattened state, leading to no measurable size change.

#### 2.2.2. The Combination of CRT with BM-MSCs-Exo Pre-Treatment Showed an Enhancing Effect on HeLa but Not on SiHa Spheroids

**Figure 4:**
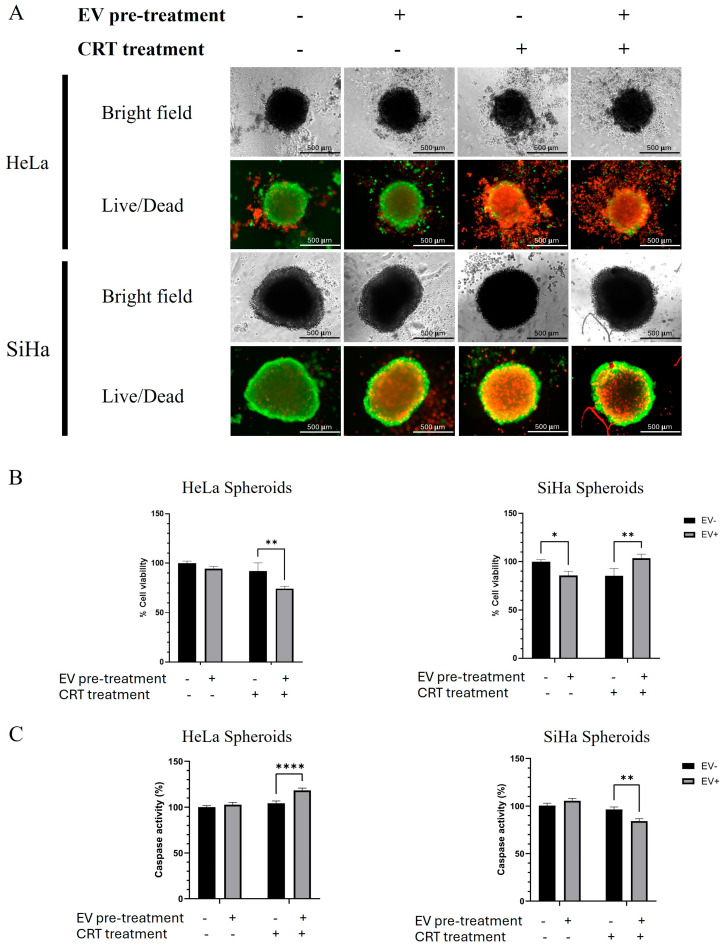
Spheroid viability and caspase activity were assessed using the LIVE/DEAD^®^ Cell Imaging Kit and ApoLive-Glo™ Multiplex Assay (**A**). The upper panel shows the cell morphology and fluorescent signals of live cells (green fluorescence) and dead cells (red fluorescence). The lower panel presents the percentage of cell viability and caspase activity in cervical cancer spheroids, (**B**) HeLa and (**C**) SiHa. The images were captured using an inverted microscope and a LionheartFX live cell imager at 4× magnification (scale bar = 500 µm). The results are presented as the mean ± SD from three independent experiments. Student’s *t*-test was used to determine statistical significance (* *p* < 0.05; ** *p* < 0.01; **** *p* < 0.0001). EV: Extracellular vesicle; CRT: Chemoradiotherapy.

After the combination of CRT treatment with BM-MSCs-exo pre-treatment, the LIVE/DEAD^®^ Cell Imaging Kit (Thermo Fisher Scientific, Waltham, MA, USA) was used to assess cell viability and death through dual fluorescent staining. Images were captured using a LionheartFX live cell imager at 4× magnification. Live cells and dead cells were distinguished by green and red fluorescence signals, respectively. Interestingly, we observed an increase in red fluorescence in the CRT-treated groups for both spheroids (Figure 4A). However, the BM-MSCs-exo pre-treatment in the CRT group of SiHa spheroids resulted in a decrease in red fluorescence compared to that in the CRT group without BM-MSCs-exo. Additionally, we observed an increase in red fluorescence in the control group of SiHa spheroids when treated with BM-MSCs-exo alone. These results suggest that BM-MSCs-exo may play a dual role in modulating cell death and are capable of both promoting and inhibiting apoptosis depending on the treatment context.

To confirm the results of the Live/Dead assay, spheroid viability and caspase activity were assessed using the ApoLive-Glo™ Multiplex Assay (Promega, USA), following the manufacturer’s instructions. The viability test results demonstrated that cell viability decreased in both spheroid types after CRT treatment (Figure 4B). Notably, BM-MSCs-exo pre-treatment in the CRT group led to a significant decrease in viability in the HeLa group, whereas it resulted in a significant increase in viability in the SiHa group.

Caspase activity strongly supported the cell viability results; an increase was observed in the CRT group with BM-MSCs-exo pre-treatment in HeLa spheroids, whereas a significant decrease was found in the same treatment group in SiHa spheroids.

Taken together, the results reveal that the BM-MSCs-exo pre-treatment increased cell death in the control group of SiHa spheroids and in the CRT group of HeLa spheroids. On the other hand, the pre-treatment decreased cell death in the CRT group of SiHa spheroids. These findings suggest that BM-MSCs-exo pre-treatment may reduce tumor spheroid sensitivity and promote chemoradiotherapy resistance in SiHa spheroids. Conversely, in HeLa spheroids, BM-MSCs-exo pre-treatment may enhance the effectiveness of CRT.

#### 2.2.3. Pre-Treatment with BM-MSCs-Exo Enhanced the CRT Treatment of HeLa Spheroids Through the Activation of Apoptotic Molecules

**Figure 5:**
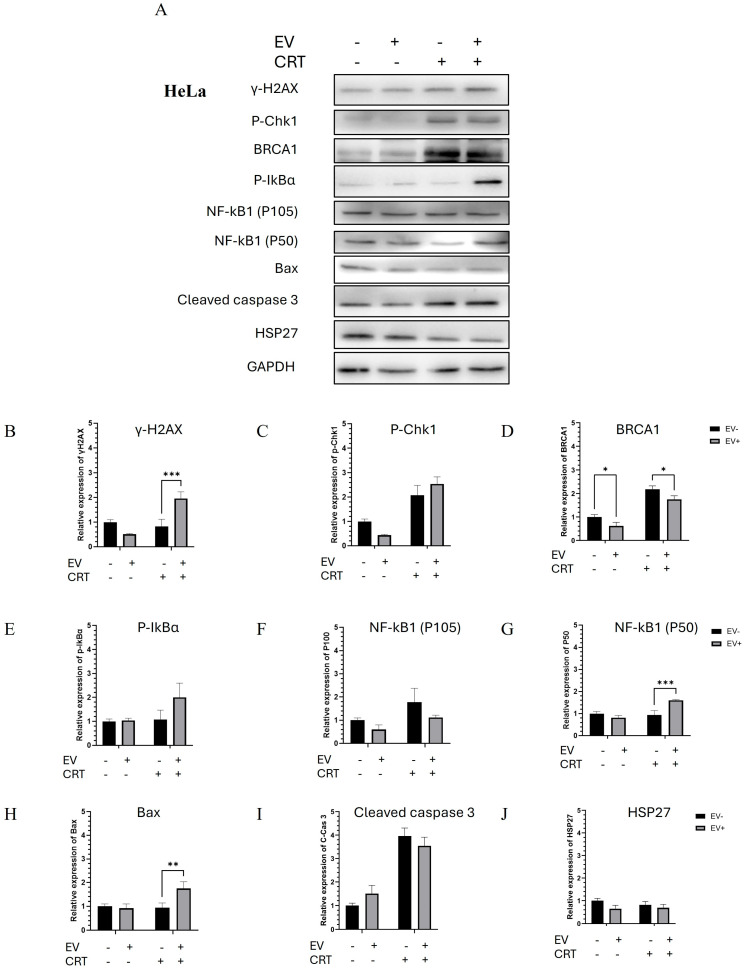
Molecular mechanisms by which BM-MSCs-exo enhanced the effect of CRT treatment on HeLa spheroids through the activation of apoptotic pathways (**A**). The expression levels of proteins involved in DNA damage, NF-κB signaling, and apoptosis pathways. The relative expression of each protein was normalized to GAPDH. Quantified proteins of interest included the following: γ-H2AX (**B**), p-Chk1 (**C**), p-BRCA1 (**D**), p-IκBα (**E**), NF-κB1 (P100) (**F**), NF-κB1 (P50) (**G**), Bax (**H**), cleaved caspase-3 (**I**), and HSP27 (**J**). Data are presented as the mean ± SD from three independent experiments. Student’s *t*-test was used to determine statistical significance (* *p* < 0.05; ** *p* < 0.01; *** *p* < 0.001). EV: Extracellular vesicle; CRT: Chemoradiotherapy.

**Figure 6:**
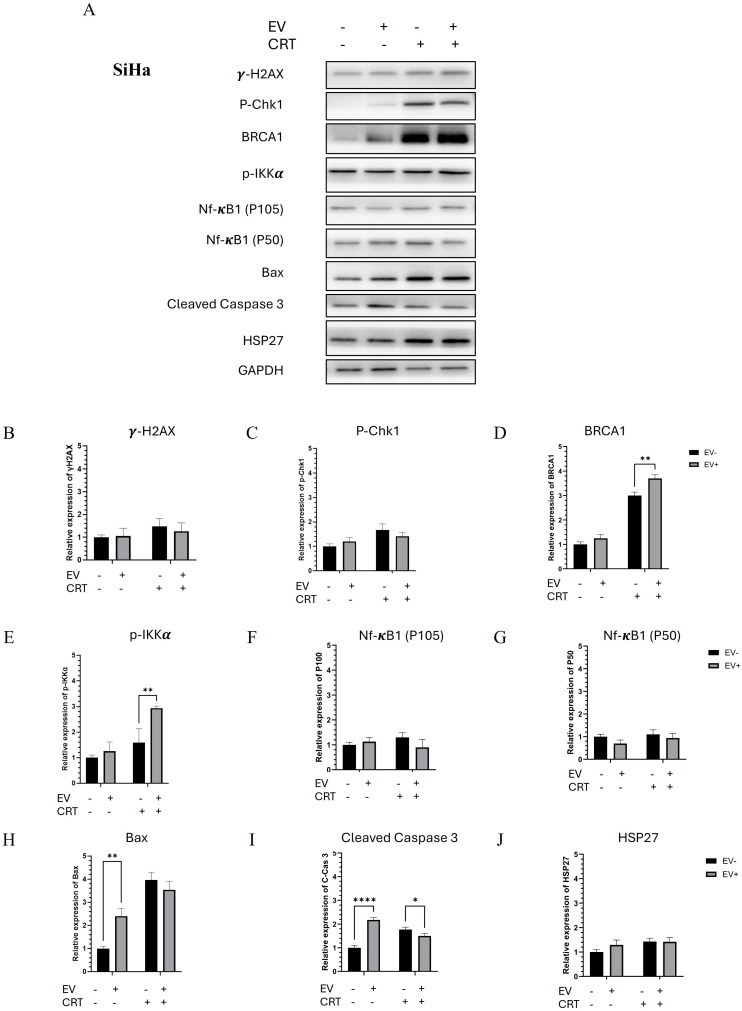
Molecular mechanisms by which BM-MSCs-exo enhanced CRT resistance in SiHa spheroids through the downregulation of NF-κB and apoptotic molecules (**A**). The expression levels of proteins involved in DNA damage, NF-κB signaling, and apoptosis pathways. The relative expression of each protein was normalized to GAPDH. Quantified proteins of interest included γ-H2AX (**B**), p-Chk1 (**C**), p-BRCA1 (**D**), p-IκBα (**E**), NF-κB1 (P100) (**F**), NF-κB1 (P50) (**G**), Bax (**H**), cleaved caspase-3 (**I**), and HSP27 (**J**), with all intensities normalized to GAPDH band intensity. Data are presented as the mean ± SD from three independent experiments. Student’s *t*-test was used to assess statistical significance (* *p* < 0.05; ** *p* < 0.01; **** *p* < 0.0001). EV: Extracellular vesicle; CRT: Chemoradiotherapy.

There was a significant increase in DNA damage-related proteins, including γ-H2AX, P-Chk1, and BRCA1, in the CRT treatment group—particularly in the BM-MSCs-exo pre-treatment group. The upregulation of these DNA damage markers led to the activation of the NF-κB pathway. An increase in P-IκBα was observed, which promoted the upregulation and nuclear translocation of the NF-κB1 (P50) transcription factor. This subsequently induced the expression of apoptotic molecules, including Bax and cleaved caspase-3. Additionally, there was a decrease in HSP27, an apoptosis-inhibiting molecule, which correlated with the increased expression of Bax. Taken together, the molecular findings confirmed the upregulation of apoptotic pathways, which correlated with the increased dead-cell population observed in the Live/Dead assay. In this context, BM-MSCs-exo pre-treatment enhanced the sensitivity of HeLa spheroids to chemoradiotherapy and promoted cell death (Figure 5 and Figure 6).

#### 2.2.4. Pre-Treatment with BM-MSCs-Exo Enhanced the Resistance of SiHa Spheroids to CRT by Downregulating NF-κB and Apoptotic Molecules

Western blot analysis revealed an increase in DNA damage-related molecules, including γ-H2AX, P-Chk1, and BRCA1, in the CRT treatment group. Interestingly, the BM-MSCs-exo pre-treatment resulted in a decreasing trend in γ-H2AX and P-Chk1 levels. P-IκBα was not detected; however, P-IKKα, a regulatory molecule of the NF-κB pathway, was strongly upregulated. The transcription factor NF-κB1 (P50) did not show a significant difference in expression. Additionally, HSP27 levels showed an increasing trend following the BM-MSCs-exo pre-treatment, which was associated with the inhibition of apoptotic molecules, including Bax and cleaved caspase-3. These results suggest that BM-MSCs-exo pre-treatment may reduce the effectiveness of CRT in SiHa spheroids by suppressing apoptosis, potentially contributing to treatment resistance.

## 3. Discussion

The successful isolation and characterization of BM-MSCs-exo were confirmed through a combination of TEM, Western blotting, and NTA. TEM imaging revealed the classical cup-shaped morphology typical of exosomes, consistent with previous descriptions of EVs in the literature [18]. The Western blot results demonstrated strong expression of exosomal markers CD63 and CD9 in the EV fraction, with minimal to no expression in the corresponding cell lysate. The absence of Cytochrome C, a mitochondrial marker, in the EV fraction further validated the purity of the exosome preparation and the absence of contamination from intracellular components [19]. NTA analysis provided quantitative insights into particle size and distribution. The particle sizes ranged from 100 to 500 nm, with a mean size of 182.9 ± 2.5 nm and a mode size of 110.0 ± 4.6 nm. This discrepancy between mean and mode particle sizes is a well-recognized phenomenon in EV research and reflects the intrinsic heterogeneity of exosome populations. The mode size is representative of the predominant exosome subset, while the elevated mean value likely results from the presence of a minority of larger vesicles, such as microvesicles or aggregates, which skew the average upward. This polydispersity is characteristic of biologically derived EV preparations and highlights the need for reporting both mean and mode values to accurately reflect the vesicle population [20,21,22]. Collectively, these findings confirm that the isolated particles are bona fide exosomes, suitable for subsequent functional analyses.

BM-MSCs-exo have previously shown potential in promoting chemoradiotherapy resistance in various types of cancer. In this study, we aimed to investigate the effect of CRT with and without BM-MSCs-exo pre-treatment in cervical cancer HeLa and SiHa spheroids. Our findings demonstrate that BM-MSCs-exo exerted differential effects depending on the cell line. The HeLa spheroids, formed over three days and containing human papillomavirus 18 (HPV-18) sequences [17], represented adenocarcinoma with low p53 expression. In this model, CRT combined with the BM-MSCs-exo pre-treatment led to apoptosis induction, as evidenced by the decrease trend in HSP27 and increase in Bax. We also observed a significant increase in γ-H2AX in the BM-MSCs-exo pre-treatment group. H2AX is a key component of the ATM complex, participating in the activation of ATM-dependent cell cycle checkpoints following double-strand breaks (DSBs) [23,24]. After radiation exposure, γ-H2AX forms nuclear foci at DSB sites, playing a crucial role in recruiting DNA repair proteins [25]. Furthermore, DSBs induced by CRT are known to activate NF-κB, which regulates both pro- and anti-apoptotic transcription [26]. Our results suggest that the increase in apoptotic cells in HeLa spheroids was a result of γ-H2AX, NF-κB1, and Bax activation.

In contrast, the SiHa spheroids, which were formed over seven days and contained HPV-16 sequences representing squamous cell carcinoma [17], showed decreased cell death and increased viability following CRT with BM-MSCs-exo pre-treatment. The molecular mechanism analysis revealed a significant increase in BRCA1 expression in the CRT+BM-MSCs-exo group. BRCA1 (Breast Cancer Susceptibility Gene 1) is a tumor suppressor involved in cell cycle control, DNA repair, checkpoint activation, transcriptional regulation, and apoptosis [27,28]. HPV oncoproteins E6 and E7 have been shown to interact with BRCA1, potentially altering its function [29]. A previous study of cervical squamous cell carcinoma (CSCC) suggested that BRCA1 overexpression may have been associated with resistance to concurrent chemoradiotherapy (CCRT) in a subset of patients [30]. In our study, the increased expression of BRCA1, along with the decreasing trends of NF-κB1, Bax, and cleaved caspase-3, may explain the survival advantage observed in SiHa spheroids.

MSC-derived exosomes contain biomolecules that mediate drug resistance, including transmembrane proteins, intracellular proteins, and microRNAs (miRNAs). These components serve as carriers of resistance mechanisms across various cancers. In breast cancer, BM-MSCs-exo have been shown to promote tumor growth both in vitro and in vivo [31]. miRNA sequencing of BM-MSCs-exo has identified high expressions of miR-30b, which plays key roles in cell proliferation, differentiation, and apoptosis depending on its targets. BM-MSCs-exo could increase the expression of Bcl-2 and decrease the expression of Bax and cleaved caspase-3, which indicated a significant anti-apoptotic effect in a mouse pulmonary microvascular endothelial cell. This evidence indicated that BM-MSCs-exo may show potential in inhibiting apoptosis via the transfer of miR30b [32]. Additionally, miR30b also acted as an apoptosis inhibitor through the targeting of caspase 3 in a glioblastoma model and EGFR in a non-small-cell lung cancer model, leading to apoptosis resistance and drug resistance [33]. Nevertheless, BM-MSCs-exo which carried miR-30b-5p showed potential in decreasing EZH2 expression, inhibiting tumor cell proliferation, migration, and invasion, and inducing apoptosis, thereby restraining the development of tumors in a non-small-cell lung cancer model [34].

Prior studies have shown that miR-30b-5p promotes apoptosis and inhibits tumorigenesis in non-small-cell lung cancer via the EZH2 and PI3K/Akt pathways [35]. BM-MSCs-exo have been proposed in apoptotic regulation resulting from a decrease in BCL and SIRT1 protein leading to the suppression of cancer cell growth and apoptosis induction. Angiogenesis suppression was evaluated in a breast cancer study through BM-MSCs-exo carrying miR221-3p, miR-16, miR-100, and miR-23b, leading to the downregulation of VEGF. This key research showed the potential of using BM-MSCs-exo in therapeutic drug delivery enhancing cancer treatment [36]. A study of ovarian cancer demonstrated that BM-MSCs-exo showed potential in inhibiting tumor growth in vitro and in vivo through the downregulation of genes in cell cycle progression [37,38]. Moreover, the overexpression of miR-199a-3p enhances the cisplatin sensitivity of ovarian cancer cells through downregulating ITGB8 expression, leading to cell cycle arrest and increased cell apoptosis. Previous studies of chemoresistant ovarian cancer tissues showed that miR-199a-3p is dysregulated and may serve as a tumor suppressor in cancer development [39].

In addition, miR-30b has been implicated in inducing apoptosis in pulmonary microvascular endothelial cells through Wnt5a and Bcl6 [32]. However, the role of these miR in cervical cancer remains unclear, and further studies are needed to elucidate the relevant proteins and pathways.

In conclusion, our results demonstrate that the effect of BM-MSCs-exo on CRT is highly dependent on tumor context, likely influenced by factors such as HPV genotype, histological subtype, and intrinsic DNA repair capacity, particularly BRCA1 activity. These findings offer novel insights into the dual role of BM-MSCs-exo and highlight the importance of precision medicine approaches in developing exosome-based therapies.

## 4. Materials and Methods

### 4.1. Cell Culture and Exosome Isolation

The BM-MSCs were kindly provided by Associate Professor Somchai Chutipongtanate, Board Certificate in Pediatrics: Ramathibodi Hospital, Mahidol University, Thailand, and Environmental & Public Health Sciences, University of Cincinnati College of Medicine, USA. To characterize the BM-MSCs, flow cytometric immunophenotyping CD14 and CD44 was used. The human mesenchymal stem cells (hMSCs) did not express hematopoietic lineage markers, such as CD14, while exhibiting positive expression for CD44, which demonstrated a characteristic immunophenotype of hMSCs.

The BM-MSCs were maintained in Dulbecco’s Modified Eagle’s Medium (DMEM, Gibco™ Thermo Fisher Scientific, Waltham, MA, USA) supplemented with 10% heat-inactivated fetal bovine serum (FBS) (Gibco™ Thermo Fisher Scientific, USA) and 1% penicillin/streptomycin (Gibco™ Thermo Fisher Scientific, Waltham, MA, USA) in a humidified incubator with 5% CO_2_ and 95% air at 37 °C. The BM-MSCs were characterized by cell surface marker staining CD14-APC (Cat. No. 561708 BD Biosciences, San Jose, CA, USA) and CD44-PE (Cat. No. 550989, BD Biosciences, San Jose, CA, USA) using the Image Stream X Mark II Imaging Flow Cytometer (Merck, Rahway, NJ, USA).

For BM-MSCs-exo isolation, cells were seeded at 80% confluency in 10 cm culture dishes. They were cultured in DMEM supplemented with 10% EV-depleted exosome FBS, as previously described [31]. After 48 h of incubation, the culture medium was collected, each batch was pooled together, and size exclusion chromatography (SEC) followed by ultracentrifugation was performed. In brief, the culture medium was centrifuged at 800× *g* for 10 min, 2000× *g* for 10 min, and 10,000× *g* for 30 min to remove cell debris. The supernatant was then concentrated using a 10 kDa Amicon^®^ Ultra Centrifugal Filter (Merck, Rahway, NJ, USA). The concentrated protein was collected, and exosomes were isolated using a qEV original 70 nm SEC column (Izon, Christchurch, New Zealand). Fractions 1–4 from the SEC column were pooled and further ultracentrifuged at 110,000× *g* for 2 h. The resulting BM-MSCs-exo pellet was resuspended in phosphate-buffered saline (PBS) for further experiments. Figure 1 presents the experimental design and workflow.

### 4.2. BM-MSC-Exo Characterization

The presence of BM-MSCs-exo was confirmed according to the MISEV protocol, using transmission electron microscopy (TEM), Western blotting, and nanoparticle tracking analysis (NTA) to validate the presence of extracellular vesicles (EVs) in the isolates [40,41].

#### 4.2.1. BM-MSC-Exo Visualization Using TEM

The protocol for electron microscopic examination was previously described [41,42]. Briefly, the isolates were fixed in 2.5% glutaraldehyde and then loaded onto carbon-coated copper grids. The grids were rinsed with DPBS, followed by distilled water. Prior to TEM imaging, the grids were stained with 1% uranyl acetate. The exosomes were visualized using a JEM-2101 transmission electron microscope (Thermo Fisher Scientific, Waltham, MA, USA).

#### 4.2.2. Western Blot Analysis of Exosome Markers

To evaluate the presence of BM-MSCs-exo, Western blot analysis was performed. BM-MSCs-exo were collected after isolation. A total of 10 µg of protein was subjected to SDS-polyacrylamide gel electrophoresis (SDS-PAGE), followed by transfer to polyvinylidene difluoride (PVDF) membranes (Amersham Pharmacia Biotech, Piscataway, NJ, USA). The membranes were blocked with 5% non-fat milk in Tris-buffered saline containing 0.1% (*v*/*v*) Tween-20 (TBS-T) for 1 h at room temperature and then washed twice with TBS-T. Each membrane was incubated overnight at 4 °C with continuous shaking in the presence of primary antibodies (diluted 1:1000 in 1% non-fat milk in TBS-T), followed by incubation with secondary antibodies specific to the primary antibodies. The list of primary and secondary antibodies used is provided in Table A1. CD63 and CD9 were used as positive markers for EVs, while Cytochrome C served as a negative marker, confirming the absence of contamination from co-isolated cellular debris. Band intensities were visualized using a Chemiluminescence & Epi Fluorescence Alliance Q9 Advanced imager (Uvitec, Cambridge, UK).

#### 4.2.3. Concentration and Size Distribution of Exosomes Evaluated Using NTA

NTA was used to evaluate the particle concentration and size distribution of BM-MSCs-exo using the NanoSight NS300 (Malvern Panalytical Ltd., Malvern, UK), as previously described [32,33]. Samples were diluted in sterile water, with two independent dilutions adjusted to approximately 20–100 particles per frame. The diluted samples were infused into the NTA analyzer using a syringe pump at a speed setting of 50. Each replicate was recorded in five 30 s videos at a controlled temperature of 25 °C, with a camera level of 14 and a detection threshold of 4. Data were analyzed using NanoSight NTA 3.0 software.

### 4.3. Pre-Treatment with BM-MSCs-Exo Followed by CRT Treatment in Cervical Cancer Spheroids

The spheroids were incubated with 10 million particles per cell or 1.56 µg of BM-MSCs-exo protein for 24 h prior to CRT treatment. Cisplatin was added to the complete medium, and this was followed by radiation treatment. The dose of cisplatin and the radiation process are detailed in Supplementary Figure S1A,B. Radiation was performed using the TrueBEAM STx platform (Varian, Palo Alto, CA, USA). Due to the greater aggression of SiHa cells, the concentration of SiHa spheroids was higher than that of HeLa spheroids. The cisplatin dose used was 0.25× IC_50_, which was 1.52 µg/mL for the HeLa and 4.36 µg/mL for the SiHa spheroids, while the radiation dose was 7 Gy (672 MV) for the HeLa and 13 Gy (1248 MV) for the SiHa spheroids. Following CRT treatment, the spheroid cells were incubated for 72 h, after which spheroid size, cell viability, caspase activity, and molecular mechanisms related to treatment response were evaluated. We hypothesized that BM-MSCs-exo exert cell line-specific effects on CRT response in cervical cancer spheroids.

### 4.4. Viability and Caspase Activity Test

Cell viability and caspase activity after CRT treatment, with and without BM-MSCs-exo pre-treatment, were evaluated using the LIVE/DEAD^®^ Cell Imaging Kit (Thermo Fisher Scientific, USA) and the ApoLive-Glo™ Multiplex Assay (Promega, Madison, WI, USA). Briefly, HeLa and SiHa cervical cancer spheroids were seeded into 96-well U-bottom Poly-HEMA-coated plates and cultured for 3 days (HeLa) and 7 days (SiHa) to allow spheroid formation. We used three spheroids per condition in each group across three independent experiments. The spheroids were then incubated with or without BM-MSCs-exo for 24 h. After incubation, the culture media were removed, and CRT treatment was applied as described above. The spheroids were further incubated for 72 h, after which spheroid size, cell viability, and caspase activity were assessed. For spheroid size analysis, images were captured using an inverted microscope at 10× magnification, and spheroid diameters were measured using ImageJ software. Statistical analysis was performed using Student’s *t*-test. For the LIVE/DEAD^®^ Cell Imaging assay, cells were processed according to the manufacturer’s protocol, and fluorescence signals were detected using a Lionheart FX Automated Microscope (BioTek, Santa Clara, CA, USA). For the ApoLive-Glo™ Multiplex Assay, fluorescence and luminescence intensities were measured using a Varioskan LUX Multimode Microplate Reader (Thermo Fisher Scientific, USA). The ApoLive-Glo kit contained a fluorogenic membrane-permeant substrate which could be cleaved by living-cell protease, producing a fluorescent signal. On the other hand, a Caspase3/7 luminogenic substrate was present, which could interact with dead cells and produce signals from luciferase reactions. Data analysis was performed using SkanIt Software for Microplate Readers RE, version 6.0.1.6.

### 4.5. Molecular Activities of BM-MSCs-Exo Pre-Treatment Combined with CRT Treatment and Western Blot Analysis

Cervical cancer spheroids, collected after CRT treatment with and without BM-MSCs-exo pre-treatment, were subjected to Western blot analysis for functional protein expression profiling. Proteins were extracted from the spheroids using RIPA buffer, and 20 µg of protein was separated via SDS polyacrylamide gel electrophoresis (SDS-PAGE), followed by transfer to polyvinylidene difluoride (PVDF) membranes (Amersham Pharmacia Biotech, USA). The membranes were blocked with 5% non-fat milk in Tris-buffered saline with 0.1% (*v*/*v*) Tween-20 (TBS-T) for 1 h at room temperature and then washed twice with TBS-T. Each membrane was incubated overnight at 4 °C, with continuous shaking, using primary antibodies (diluted 1:1000 in 1% non-fat milk in TBS-T) specific to the target proteins. This was followed by incubation with secondary antibodies (also diluted 1:1000 in 1% non-fat milk in TBS-T) specific to the primary antibodies. The list of primary and secondary antibodies is provided in Table A1, and GAPDH was used as the internal loading control. Protein bands were detected using Pierce™ ECL Western blotting Substrate (Thermo Fisher Scientific, USA) and visualized with the Chemiluminescence & Epi Fluorescence Alliance Q9 Advanced imager (Uvitec, UK). Band intensities were quantified using the same imaging system, with GAPDH normalization.

## 5. Conclusions

**Figure 7:**
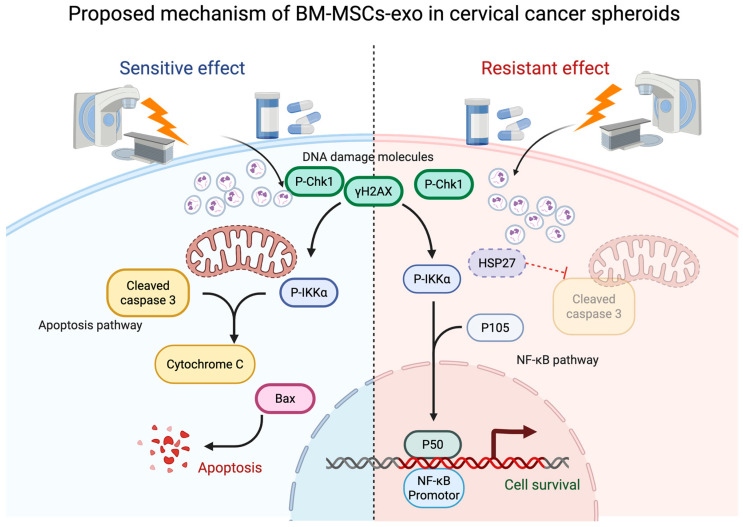
Proposed molecular mechanism of BM-MSC-derived exosomes (BM-MSCs-exo), illustrating their dual role in promoting apoptosis and contributing to chemoradiotherapy (CRT) resistance in cervical cancer spheroids. The diagram highlights NF-κB activation and its downstream signaling pathways as potential mediators of these effects. Experimentally validated mechanisms are indicated by solid arrows, whereas hypothetical or inferred interactions are represented by dashed arrows. Figure created with BioRender. Nittayaboon, K. (2025).

To conclude, Figure 7 illustrates the proposed mechanism by which BM-MSCs-exo functioned either as a CRT sensitizer or a resistance inducer in cervical cancer spheroids. The primary mechanism by which CRT induced apoptosis was DNA damage. In the CRT sensitizer mode, DNA damage-related molecules were upregulated, activating the NF-κB pathway, which in turn led to the activation of apoptosis-related proteins, ultimately resulting in increased apoptosis and cell death. On the other hand, in the CRT resistance mode, although DNA damage markers were also increased, the NF-κB transcription factor and the apoptosis inhibitor HSP27 showed an increasing trend, which led to the downregulation of apoptotic molecules. This resulted in reduced cell death and increased cell viability.

## Data Availability

The data presented in this study are available on request from the corresponding author. The data are not publicly available due to restrictions imposed by collaborative research agreements.

## Supplementary Materials

The following supporting information can be downloaded at https://www.mdpi.com/article/10.3390/ph18071050/s1. Figure S1: The whole membrane of all 3 replicates of Western blotting. Figure S2: The quantification of HeLa (A) and SiHa (B) spheroids in all 3 replicates of Western blotting. GAPDH was used as a reference protein for band intensity. Figure S3: The percentage of cell viability after the radiation treatment of HeLa (A) and SiHa (B) spheroids. Figure S4: The percentage of cell viability after the cisplatin treatment of HeLa (A) and SiHa (B) spheroids. Table S1: The protein intensity of HeLa spheroids after CRT treatment with or without BM-MSCs-exo pre-treatment. Table S2: The protein intensity of SiHa spheroids after CRT treatment with or without BM-MSCs-exo pre-treatment. Table S3: The IC50 of the radiation treatment. Table S4: The IC50 of the cisplatin treatment.

## Abbreviations

The following abbreviations are used in this manuscript:

CSCC	cervical squamous cell carcinoma
HPV	human papilloma virus
BM-MSCs	bone marrow mesenchymal stem cells
BM-MSC-exo	bone marrow mesenchymal stem cell-derived exosome
MSCs	mesenchymal stem cells
MSC-exo	mesenchymal stem cell-derived exosome
CRT	chemoradiotherapy
DSBs	double-strand breaks
SDS-PAGE	SDS polyacrylamide gel electrophoresis
NTA	nanoparticle tracking analysis
TEM	transmission electron microscope
PVDF membranes	polyvinylidene difluoride membranes
TBS-T	Tris-buffered saline with 0.1% (*v*/*v*) Tween-20
PBS	phosphate-buffered saline

## Appendix A

**Table A1:** List of Antibody used in the experiments.

Name of Antibody (Cat No.)	Molecular Weight (kDa)	Dilution
Antibody for BM-MSCs-exo		
CD9 Rabbit mAb (13174)	22–24	1:1000
CD63 Rabbit mAb (52090)	25–60	1:1000
β-Actin Antibody (4967)	45	1:1000
Cytochrome c Rabbit mAb (11940)	14	1:1000
Antibody for molecular mechanism		
DNA Damage Pathway		
Phospho-BRCA1 (Ser1524) Antibody (9009)	220	1:1000
Phospho-Chk1 (Ser345) Rabbit mAb (2348)	56	1:1000
Phospho-Histone H2A.X (Ser139) Rabbit mAb (9718)	15	1:1000
NF-κB Pathway		
Phospho-IKKα/β (Ser176/180) Rabbit mAb (2697)	85	1:1000
Phospho-IκBα (Ser32) Rabbit mAb (2859)	40	1:1000
NF-κB1 p105/p50 Rabbit mAb (13586)	50 (Active Form) 120 (Precursor)	1:1000
Apoptosis Pathway		
Cleaved Caspase-3 (Asp175) Rabbit mAb (9664)	19	1:1000
Bax (D2E11) Rabbit mAb (5023)	20	1:1000
Phospho-HSP27 (Ser82) Rabbit mAb (9709)	27	1:1000
Internal Control		
GAPDH Rabbit mAb (2118)	37	1:1000
Secondary Antibody		
Anti-rabbit IgG, HRP-linked Antibody (7074)		1:1000
