# Supplementary material for: Bone Marrow Mesenchymal Stem Cell-Derived Exosomes Modulate Chemoradiotherapy Response in Cervical Cancer Spheroids
Source: Pharmaceuticals (Basel). 2025 Jul 17;18(7):1050. doi: 10.3390/ph18071050 (PMC12299787; doi:10.3390/ph18071050)
Supplement: Supplementary file 1 [file pharmaceuticals-18-01050-s001.zip › pharmaceuticals-3748986-supplementary.pdf]

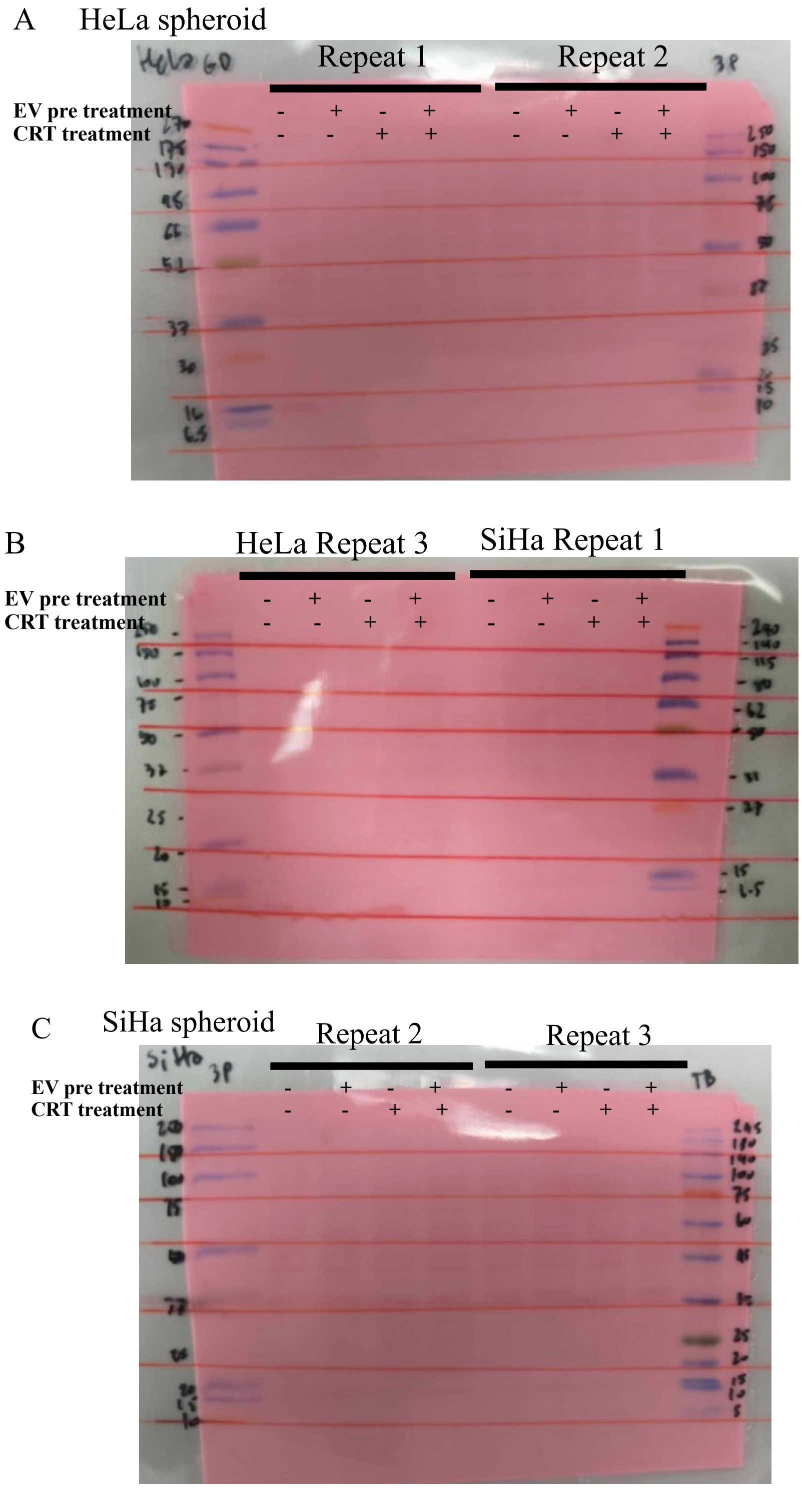

Figure S1. The whole membrane of all 3 replicates of Western Blotting.

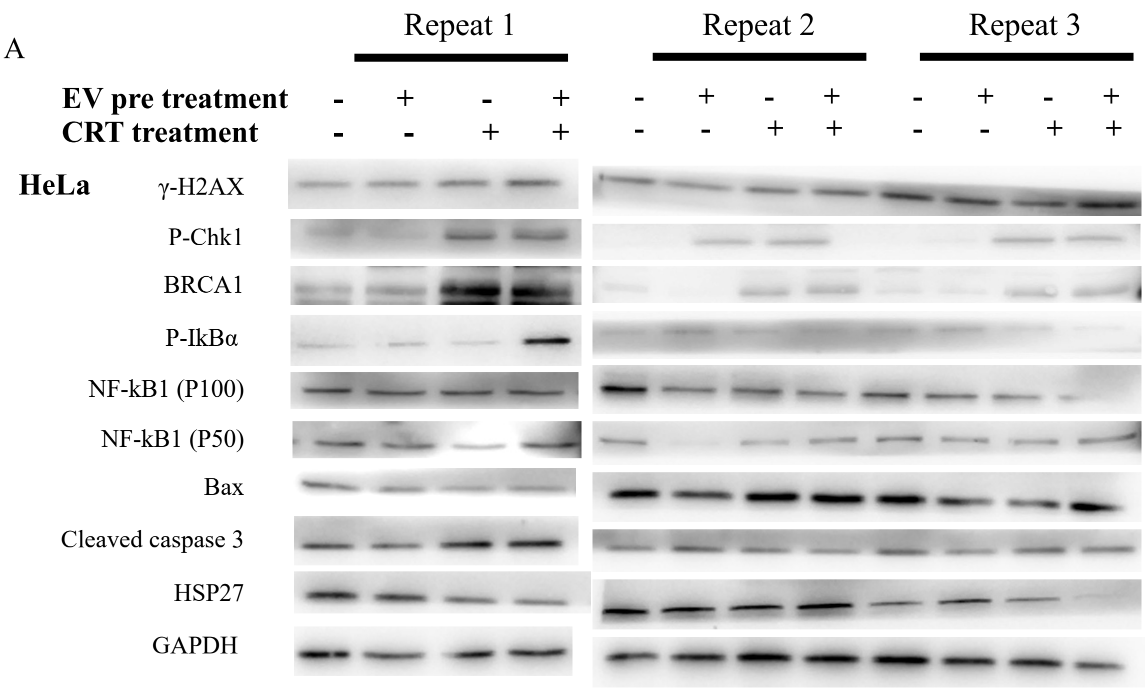

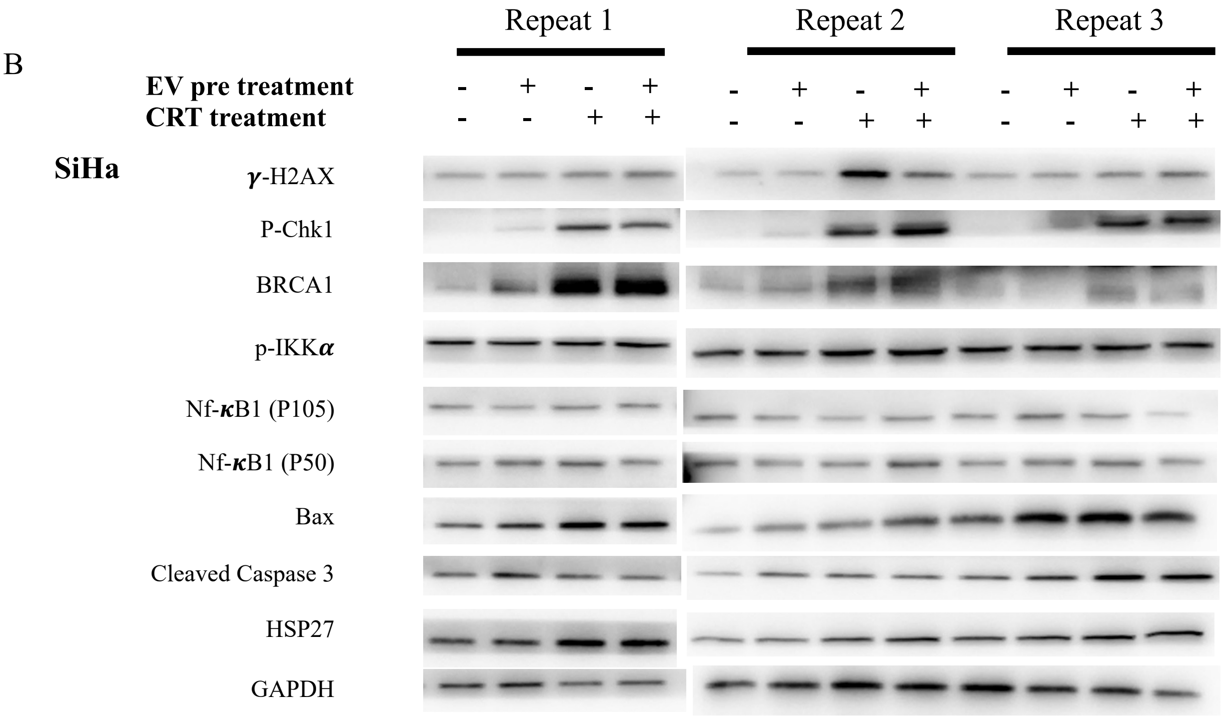

Figure S2. The quantification of HeLa (A) and SiHa (B) spheroids in all 3 replicates of Western Blotting. GAPDH was used as a reference protein for band intensity.

Table S1. The protein intensity of HeLa spheroids after CRT treatment with or without BM-MSCs-exo pre-treatment.

HeLa	Repeat 1				Repeat 2				Repeat 3			
EV pretreatment	-	-	+	+	-	-	+	+	-	-	+	+
CRT treatment	-	+	-	+	-	+	-	+	-	+	-	+
H2AX	175,477	123,058	195,927	160,266	166,160	160,265	156,518	190,854	256,677	284,141	297,906	395,871
p-Chk1	187,662	167,469	221,987	245,666	29,407	23,899	64,858	41,731	94,126	67,198	93,118	101,713
BRCA1	182,820	146,511	324,437	313,608	122,749	240,372	383,965	300,798	45,243	69,965	110,683	118,846
p-IkBα	277,377	261,373	251,297	317,000	67,266	101,431	127,131	182,849	81,547	46,716	50,204	61,934
NF-$\boldsymbol{\kappa}$B (P105)	223,777	163,492	182,798	147,636	169,860	135,272	106,641	97,679	226,018	227,751	124,897	151,483
NF-$\boldsymbol{\kappa}$B (P50)	64,904	43,867	26,126	54,232	149,274	98,673	148,073	170,307	67,265	40,585	60,270	94,592
Bax	170,376	214,285	100,212	188,902	208,307	227,789	240,234	268,898	149,878	144,607	148,435	163,332
Clevaed Caspase3	184,307	148,590	122,354	126,935	105,777	128,882	121,155	119,123	255,196	210,231	265,239	317,043
HSP27	336,797	323,812	301,738	314,973	368,214	363,327	292,370	165,915	152,500	167,758	72,669	55,376
GAPDH	135,060	162,372	46,272	257,230	250,920	231,755	122,256	644,53	163,073	199,522	199,626	195,657

Table S2. The protein intensity of SiHa spheroids after CRT treatment with or without BM-MSCs-exo pre-treatment.

SiHa	Repeat 1				Repeat 2				Repeat 3			
EV pretreatment	-	-	+	+	-	-	+	+	-	-	+	+
CRT treatment	-	+	-	+	-	+	-	+	-	+	-	+
H2AX	81,094	76,518	111,284	157,372	114,534	114,341	143,900	164,843	173,492	290,369	306,075	401,748
p-Chk1	95,422	97,077	103,828	181,694	78,307	70,249	119,790	200,490	57,238	51,142	76,684	60,838
BRCA1	182,820	146,511	324,437	313,608	122,749	240,372	383,965	300,798	45,243	69,965	110,683	118,846
IKKa	120,998	137,562	162,494	189,085	152,856	171,099	191,630	224,905	143,408	138,087	158,566	264,849
NF-$\boldsymbol{\kappa}$B (P105)	223,777	163,492	135,272	106,641	147,864	156,098	122,360	191,953	112,036	134,637	146,054	136,072
NF-$\boldsymbol{\kappa}$B (P50)	64,904	43,867	53,140	55,421	149,274	98,673	84,639	181,024	53,748	22,538	40,006	30,858
Bax	317,000	261,373	251,297	277,377	297,689	235,539	207,321	265,044	159,429	245,035	275,830	402,016
Clevaed Caspase3	116,741	167,326	120,204	116,629	149,672	117,058	166,877	178,077	101,613	110,313	143,579	182,054
HSP27	125,474	243,947	176,234	204,787	251,955	239,035	190,601	140,575	68,644	70,146	65,467	85,151
GAPDH	140,661	181,300	109,206	101,711	144,127	159,523	129,472	143,129	237,761	195,657	188,264	126,079

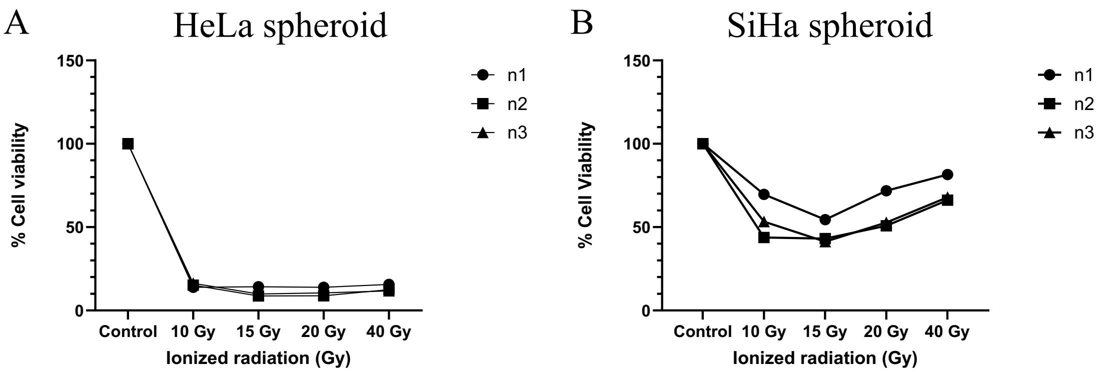

Figure S3. The percentage of cell viability after the radiation treatment of HeLa (A) and SiHa (B) spheroids.

Table S3. The IC50 of the radiation treatment

Cell type	IC50 of radiation dose (Gy)
HeLa	7
SiHa	13

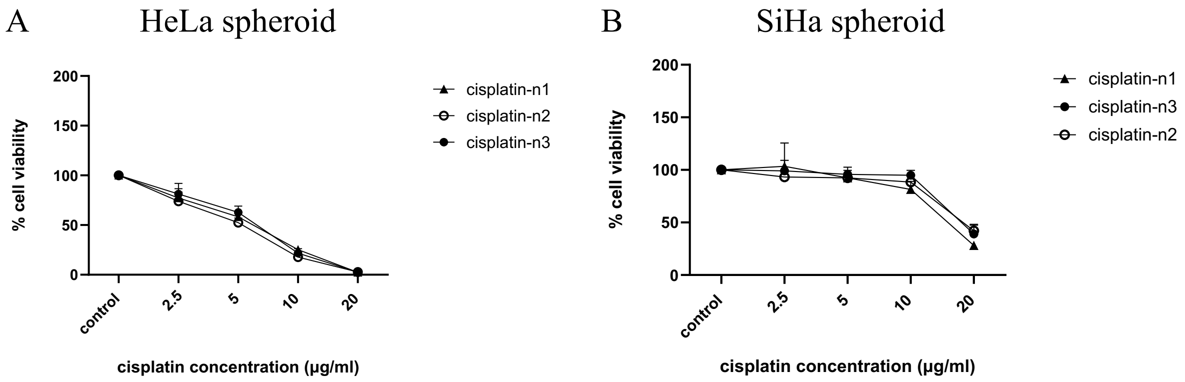

Figure S4. The percentage of cell viability after the cisplatin treatment of HeLa (A) and SiHa (B) spheroid.

Table S4. The IC50 of the cisplatin treatment

Cell type	IC50 of cisplatin dose (µg/ml, mean ± SD)
HeLa	6.05 ± 0.52
SiHa	17.45 ± 0.49
